# Isolation and Characterization of a Defensin-Like Peptide (Coprisin) from the Dung Beetle, *Copris tripartitus*

**DOI:** 10.1155/2009/136284

**Published:** 2009-10-22

**Authors:** Jae-Sam Hwang, Juneyoung Lee, Yeon-Ju Kim, Hea-Son Bang, Eun-Young Yun, Seong-Ryul Kim, Hwa-Jin Suh, Bo-Ram Kang, Sung-Hee Nam, Jae-Pil Jeon, Iksoo Kim, Dong Gun Lee

**Affiliations:** ^1^National Academy of Agricultural Science, RDA, Suwon 441-707, South Korea; ^2^School of Life Sciences and Biotechnology, College of Natural Sciences, Kyungpook National University, Daegu 702-701, South Korea; ^3^Department of Biotechnology, Daegu University, Kyungbuk 712-714, South Korea; ^4^Center for Genome Science, Korea National Institute of Health, Seoul 122-701, South Korea; ^5^College of Agriculture and Life Sciences, Chonnam National University, Gwangju 500-757, South Korea

## Abstract

The antibacterial activity of immune-related peptides, identified by a differential gene expression analysis, was investigated to suggest novel antibacterial peptides. A cDNA encoding a defensin-like peptide, Coprisin, was isolated from bacteria-immunized dung beetle, *Copris tripartitus*, by using differential dot blot hybridization. Northern blot analysis showed that Coprisin mRNA was up-regulated from 4 hours after bacteria injection and its expression level was reached a peak at 16 hours. The deduced amino acid sequence of Coprisin was composed of 80 amino acids with a predicted molecular weight of 8.6 kDa and a pI of 8.7. The amino acid sequence of mature Coprisin was found to be 79.1% and 67.4% identical to those of defensin-like peptides of *Anomala cuprea* and *Allomyrina dichotoma*, respectively. We also investigated active sequences of Coprisin by using amino acid modification. The result showed that the 9-mer peptide, LLCIALRKK-NH_2_, exhibited potent antibacterial activities against *Escherichia coli* and *Staphylococcus aureus*.

## 1. Introduction

Antibacterial peptides play important roles in the innate immune systems of vertebrates and invertebrates. The field of innate immunity in invertebrates, especially the study of particular insects, has revealed the importance of antimicrobial peptides in their defense systems. Most of the peptides are produced in the fat body or haemocytes and then released into the hemolymph of insects [1–3]. They are known to play important roles in humoral defense reactions [4–8]. In addition to defense responses in the circulatory system, antimicrobial peptides are synthesized as effector molecules in epithelial and midgut tissues, which form a critical interface from the external environment [9–11]. So far, more than 200 antibacterial peptides have been identified in insects. Based on their amino acid sequences and antibacterial activities, most of the peptides are divided into five groups including cecropin, insect-defensins, glycine-rich proteins, proline-rich proteins, and lysozymes. These peptides can be further classified into three distinct groups: linear alpha-helical peptides devoid of cysteine residues, peptides in which proline and/or glycine residues are over-represented, and cysteine-rich peptides with cysteine stabilized *α*-*β* motifs [12].

*Copris tripartitus* spends most of its time in dung, where pathogens are abundant. Their larvae feed on the fungi, decaying organic matter, dung, and other organic materials found in dung balls. Therefore, it is likely that *C. tripartitus* defends itself against invading pathogens by means of antimicrobial compounds. The purpose of the present study was to isolate and identify immune related genes in the dung beetle, *C. tripartitus*. In this study, we constructed a full-length cDNA library from bacteria-immunized *C. tripartitus* and then selected up-regulated clones using differential screening by a dot blot hybridization analysis. One of these up-regulated clones was isolated and characterized. This is the first report of the isolation and characterization of the defensin-like peptide, Coprisin, from the dung beetle, *C. tripartitus*.

## 2. Materials and Methods

### 2.1. Immunization

To induce antibacterial peptides, the *C. tripartitus* larvae were cooled on ice and individually injected with 50 *μ*L of *E. coli* JM109 (5 × 10^5^ cells) suspended in physiological saline (150 mM NaCl/5 mM KCl).

### 2.2. Differential Gene Expression and Northern Blot Analysis

As an initial screening process, a total of 1862 cDNA clones were randomly selected from a full-length cDNA library, spotted onto Hybond-N membranes (Amersham Biosciences, Uppsala, Sweden) using a 96-well format dot blotter (Bio-RAD) and then hybridized with probes from bacteria-injected larvae as described previously [13]. Coprisin cDNA clones were identified by a dot blot hybridization. Total RNA was extracted with a Trizol reagent (Life technologies, Inc., Gaithersburg, MD, USA) from the whole larvae 0, 4, 8, 16, and 24 hours after *E. coli* injection and quantified by ultraviolet (UV) spectroscopy. Northern blot analysis was performed as described previously [13]. Briefly, 10 *μ*g of total RNA was fractionated on 1% agarose/6.7% formaldehyde. Hybridization was performed for 3 hours at 65°C in the ExpressHyb hybridization solution, according to the manufacturer's instruction (Clontech). As an internal marker, 28S rRNA was visualized by ethidium bromide (EtBr) staining.

### 2.3. Peptide Synthesis

The peptides were chemically synthesized in the peptide synthesis facility, PepTron Inc. (Daejeon, Korea). These synthetic peptides were purified by reverse-phase HPLC using a capcell pak C18 column (Shiseido). Elution was performed with a water-acetonitrile linear gradient (0 ~ 80% of acetonitrile) containing 0.1% (v/v) trifluoroacetic acid (TFA). The correct identification of the peptides was confirmed by using an ESI mass spectrometer (Plaform II, Micromass, Manchester, UK) and MALDI-TOF mass spectrometer (Voyager-DESTR, Applied Biosystem).

### 2.4. Antibacterial Activity Assay

The antibacterial activity of each synthetic peptide was initially examined by the radial diffusion assay as described previously [14]. Briefly, bacteria were grown overnight in a tryptic soy broth (TSB, Difco) to the onset of stationary phase at 37°C with shaking at 200 rpm. The cultures were diluted in a fresh TSB and were incubated at 37°C until the optical density reached 0.4 at 620 nm. The cultured bacteria were centrifuged at 3000 rpm for 10 min at 4°C, washed two times in cold 10 mM sodium phosphate buffer (SPB, pH 7.4), and resuspended in cold SPB. A volume containing 4 × 10^6^ CFU bacteria was added to 10 mL of warm (40 to 50°C) citrate phosphate buffer (9 mM sodium phosphate, 1 mM sodium citrate, pH 7.4) containing 1% (w/v) low-electroendosmosis-type agarose (Sigma) and 0.03% TSB. Three-millimeter diameter holes were punched in the set agarose and filled with 5 *μ*L of test peptides. After allowing 3 hours for diffusion of the peptides, a 10 mL nutrient-rich overlay gel containing 6% TSB and 1% (w/v) agarose was overlaid and was then incubated overnight at 37°C. The diameters of clear zones surrounding each well were measured and expressed in activity units (1 mm = 10 units).

In addition to a radial diffusion assay, antibacterial activities of the peptides were also tested by a broth microdilution assay against* E. coli* and* S. aureus*. Briefly, bacteria were grown overnight in TSB to the onset of stationary phase at 37°C with shaking at 200 rpm. The cultures were diluted in a fresh TSB to the final concentration of 2 × 10^4^  CFU/mL. A stock solution of each peptide was prepared in 0.01% acetic acid at 640 *μ*g/mL. The peptide solution was then serially diluted in 0.01% acetic acid to 2 *μ*g/mL. After 90 *μ*L aliquots of the bacterial suspension were dispensed into each well of a 96-well polypropylene microtiter plate, 10 *μ*L of peptide solution was added. The antibacterial activities of peptides were assessed by measuring visible turbidity in each well of the plate after 18 hours of incubation at 37°C. MIC was expressed as intervals (a to b), where a was the highest concentration tested at which bacteria were still growing and b was the lowest concentration that caused complete growth inhibition.

## 3. Results and Discussion

**Figure 1:**
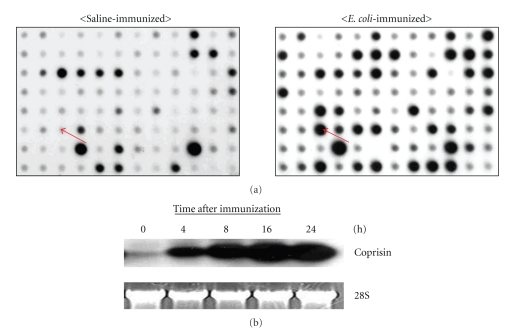
Isolation of Coprisin cDNA. (a) Differential dot blot hybridization. Randomly selected 1862 cDNA clones from cDNA library of *Copris tripartitus* larvae were hybridized with probes of *E. coli*-immunized larvae. In this figure, one pair of hybridization dot blots is shown for the Coprisin cDNA clone (adapted from Hwang et al. [13]). The arrow indicates a differentially expressed Coprisin cDNA clone in saline- or *E. coli*-immunized larvae. (b) Northern blot hybridization of the Coprisin gene for a time-course after immunization. *C. tripartitus* larvae were injected with 50 *μ*L of *E. coli* JM109 (5 × 10^5^ cells) suspended in physiological saline (150 mM NaCl/5 mM KCl). Larvae were kept for 0, 4, 8, 16, and 24 hours. Ten microgram aliquots of total RNA were resolved on formaldehyde containing agarose gels and blotted onto nitrocellulose membranes. The probe was labeled with [*α*-^32^P]dCTP. As an internal marker, 28S rRNA was stained with ethidium bromide.

In order to find antibacterial peptides responsible for bacterial resistance, we performed the differential hybridization with all of the cDNA probes, synthesized from normal and immunized larvae from a dung beetle, *C. tripartitus*. Thirteen individual cDNA transcripts were identified as differentially expressed sequences by the dot blot hybridization [13]. One of the up-regulated genes (hereinafter referred to as Coprisin) is a novel member of a family of antibacterial peptides known as insect defensin-like peptides (Figure 1(a)). To confirm the inducibility of Coprisin by immune challenge, we performed a Northern blot analysis using the total RNA extracted from the *E. coli* immunized larvae (Figure 1(b)). The immunized larvae were collected and total RNA was isolated at different time points after *E. coli* injection. The mRNA expression was up-regulated after 4 hours, and its expression level reached a peak level after 16 hours. This result suggested that the increase of Coprisin expression after the *E. coli* injection may be involved in the immune response of the dung beetle.

**Figure 2:**
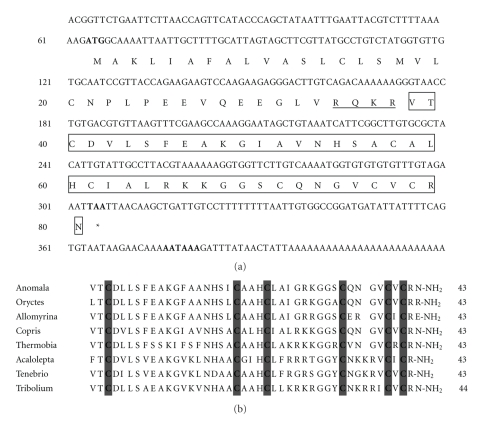
Full-length cDNA sequence and comparison of amino acid sequences of insect defensins. (a) Nucleotide and deduced amino acid sequences of Coprisin gene isolated from *C. tripartitus*. The predicted amino acid sequence (single-letter abbreviation) is shown below the nucleotide within the open reading frame (ORF). The potential recognition sequence for the cleavage site within the constitutive secretory pathway (Arg-Xaa-Lys/Arg-Arg) is single-underlined. The putative mature peptide is boxed. Asterisk indicates the termination codon. Codons for initiation, termination, polyadenylation, and poly(A) tail are in bold. (b) Comparison of seven insect defensins sequences. Bars indicate gaps to optimize the sequence alignment. The six observed Cys(C) residues, which make up the disulphide bridges, are shaded grey. The GenBank accession numbers for the analyzed sequence are *Anomala cuprea* (BAD77966), *Oryctes rhinoceros* (BAA36401), *Allomyrina dichotoma* (AAB36306), *Copris tripartitus* (ABP97087), *Thermobia domestica* (CAM36306), *Acalolepta luxuriosa* (AAK35160), *Tenebrio molitor* (BAA04552), and *Tribolium castaneum* (XP968237).

The full length cDNA sequence of Coprisin was 412 bp in length, having a 5′ untranslated region (UTR) of 63 bp, a 3′ UTR of 106 bp, and open reading frame (ORF) of 240 bp (Figure 2(a)) coding for a putative preprodefensin of 80 amino acid residues with a predicted molecular mass of 8.6 kDa and a pI of 8.72 (Figure 2(b)). The Coprisin cDNA sequence contained predicted signal peptide cleavage sites at Cys20 which produces mature 43-residue peptides resulting in a theoretical molecular mass of 4.5 kDa and a PI of 8.67. The amino acid sequence of the Coprisin precursor exhibits 62% similarity to that of the *Anomala cuprea* defensin (data not shown), whereas the mature portion of Coprisin displays 79%, 67%, and 72% similarity to those of *A. cuprea*, *Allomyrina dichotoma*, and *Oryctes rhinoceros* defensins, respectively, suggesting that Coprisin is an insect defensin (Figure 2(b)). The six cysteine residues known to form the three disulphide bridges in the defensin molecule are at positions 3, 20, 24, 34, 39, and 41.

Insect defensins are the best-known peptides with a CS*α* *β* motif, and they all have a C ⋯ CXXXC ⋯ C ⋯ CXC consensus sequence [1]. The deduced amino acid sequence of the Coprisin peptide showed that it had a cysteine-stabilized *α* *β* motif with a C ⋯ CXXXC ⋯ C ⋯ CXC consensus sequence, as found in other insect defensins. A multiple sequence alignment analysis using CLUSTALW showed that this peptide is distinct from other insect defensins, indicating that it is a novel peptide with a CS*α* *β* motif. To find the antibacterial active region of Coprisin, we synthesized four peptides that had the amidated amino acid residues at their C-terminal corresponding to amino acid residues 1V-43N-NH_2_ (CopN1), 5V-16N-NH_2_ (CopN2), 19A-30K-NH_2_ (CopN3), and 31G-43N-NH_2_ (CopN4) and then examined their antibacterial activity against* E. coli* and* S. aureus* by the radial diffusion assay. Of these peptides, CopN1 and CopN3 peptides showed strong antibacterial activity, whereas other peptides had substantially less or no antibacterial activity (data not shown). The CopN3 peptide fragment corresponded to the *α*-helical region of a known insect defensin-like peptide [15]. Truncation of three amino acids from the CopN3 peptide (resulting in CopN5) still showed potent activity which was as much as the CopN3 fragment.

To further increase antibacterial activity of CopN5, we then synthesized four 9-mer peptides modified by changing single amino acid residues of CopN5 sequence (see Table 1). In CopA1 and CopA3, cysteine and histidine residues were replaced by leucine to increase the hydrophobicity. In CopA2 and CopA4, cysteine and histidine residues were replaced by arginine to increase the electropositivity. We examined the antibacterial activities of modified peptides against *E. coli* and* S. aureus*. The results of the MIC test are shown in Table 1. Among these peptides, CopA3 exhibited the strongest activity, with MIC values in 4–8 *μ*g/mL range. These results suggest that a moderate hydrophilic-hydrophobic balance of the *α*-helical antimicrobial peptides is a crucial factor in designing novel peptides in order to increase potent antibiotic activity. The first insect defensin peptide was isolated by Matsuyama and Natori [16] from the flesh fly *Sarcophaga peregrina*. Since then, numerous insect defensins have been isolated from various insects. Peptides in the insect defensin family commonly have molecular weights of 3-4 kDa and three disulfide bridge, while they showed potent antibacterial activity against various MRSA and lesser activity against Gram negative bacteria. As shown in Figure 2(b), amino acid alignments of the insect defensin family indicated that the three-disulfide bridges are conserved in insect defensin peptides. Many insect defensin proteins have variable N-terminal loop sizes in which the number of residues ranges from 6 to 17 between the two cysteines. This suggests that this region may not be essential for antibacterial activity but contributes to specificity.

## Figures and Tables

**Table 1:** The antimicrobial activity of Coprisin peptides against *E. coli* and *S. aureus*.

Peptides	Amino acid sequence	MIC (*μ*g/mL)	
		*E. coli*	*S. aureus*
CopN5(22–30)	LHCIALRKK-NH_2_	8–16	>64
CopA1	LH**L**IALRKK-NH_2_ (C^24^ → L^24^)	>64	>64
CopA2	LH**R**IALRKK-NH_2_ (C^24^ → R^24^)	>64	>64
CopA3	L**L**CIALRKK-NH_2_ (H^23^ → L^23^)	4–8	4–8
CopA4	L**R**CIALRKK-NH_2_ (H^24^ → R^24^)	16–32	>64
